# Impact of stem design and cementation on postoperative femoral antetorsion in 227 patients with total hip arthroplasty (THA)

**DOI:** 10.1007/s00256-020-03483-z

**Published:** 2020-06-25

**Authors:** Tim Fischer, Christoph Stern, Benjamin Fritz, Patrick O. Zingg, Christian W. A. Pfirrmann, Reto Sutter

**Affiliations:** 1 Department of Radiology, Balgrist University Hospital, University of Zurich, Forchstrasse 340, 8008 Zurich, Switzerland; 2 Department of Orthopedics, Balgrist University Hospital, University of Zurich, Forchstrasse 340, 8008 Zurich, Switzerland

**Keywords:** Hip, Prosthesis, Femoral antetorsion, Cementation

## Abstract

**Objective:**

In total hip arthroplasty (THA), surgeons attempt to achieve a physiological antetorsion. However, postoperative antetorsion of the femoral stem is known to show large variabilities. The purpose of this study was to assess whether postoperative antetorsion is influenced by stem design or cementation.

**Materials and methods:**

This retrospective study included 227 patients with a hip prosthesis with five different stem designs (S1: short curved, S2 and S3: standard straight, S4: standard straight collared, S5: cemented straight), who had metal suppressed 1.5T-MRI of the hip between February 2015 and October 2019. Measurement of femoral antetorsion was done independently by two fellowship-trained radiologists on axial images by measuring the angle between the long axis of the femoral neck and the posterior condylar tangent of the knee. Measured angles in the different groups were compared using the *t* test for independent samples.

**Results:**

The cementless collared stem S4 showed the highest antetorsion with 18.1° (± 10.5°; range –10°–45°), which was significantly higher than the antetorsion of the collarless S3 with 13.3° (± 8.4°; − 4°–29°) and the cemented S5 with 12.7° (± 7.7°; − 3°–27°) with *p* = 0.012 and *p* = 0.007, respectively. S1 and S2 showed an antetorsion of 14.8° (± 10.0°; 1°–37°) and 14.1° (± 12.2°; − 20°–41°). The torsional variability of the cementless stems (S1–4) was significantly higher compared with that of the cemented S5 with a combined standard deviation of 10.5° and 7.7° (*p* = 0.019).

**Conclusion:**

Prosthesis design impacts the postoperative femoral antetorsion, with the cementless collared stem showing the highest antetorsion. Cemented stems demonstrated significantly lower variability, suggesting the lowest rate of inadvertent malrotation.

## Introduction

In many orthopedic centers, the femoral antetorsion angle is a routinely measured value since abnormal torsion is associated with a variety of disorders of the hip and knee joint. Correlation between the anatomic configuration of the femur and hip dysplasia or slipped capital femoral epiphysis was established many years ago [1, 2]; more recent findings include an association between abnormal femoral antetorsion and femoroacetabular impingement as well as patellar instability [3, 4].

In total hip arthroplasty (THA), stem antetorsion and cup alignment affect postoperative joint range of motion and are key factors related to impingement and dislocation [5–9]. Increased anteversion of the acetabular cup is associated with anterior dislocation of the THA [10], but postoperative cup alignment is quite precise since the cup can be placed into the correct position before locking it to the acetabulum.

During surgery, a femoral antetorsion of 15° is targeted [11], yet postoperative femoral antetorsion angles in patients with THA are sometimes suboptimal and are known to have a wide range [12]. There is no data available regarding the influence of stem design and cementation on femoral torsion.

We set out to evaluate whether different stem designs and the presence or absence of cementation are associated with different postoperative femoral antetorsion angles and whether these factors influence postoperative torsional variability as an indicator of how precise the preoperatively targeted antetorsion is reached postoperatively.

## Materials and methods

### Patients

**Fig. 1:**
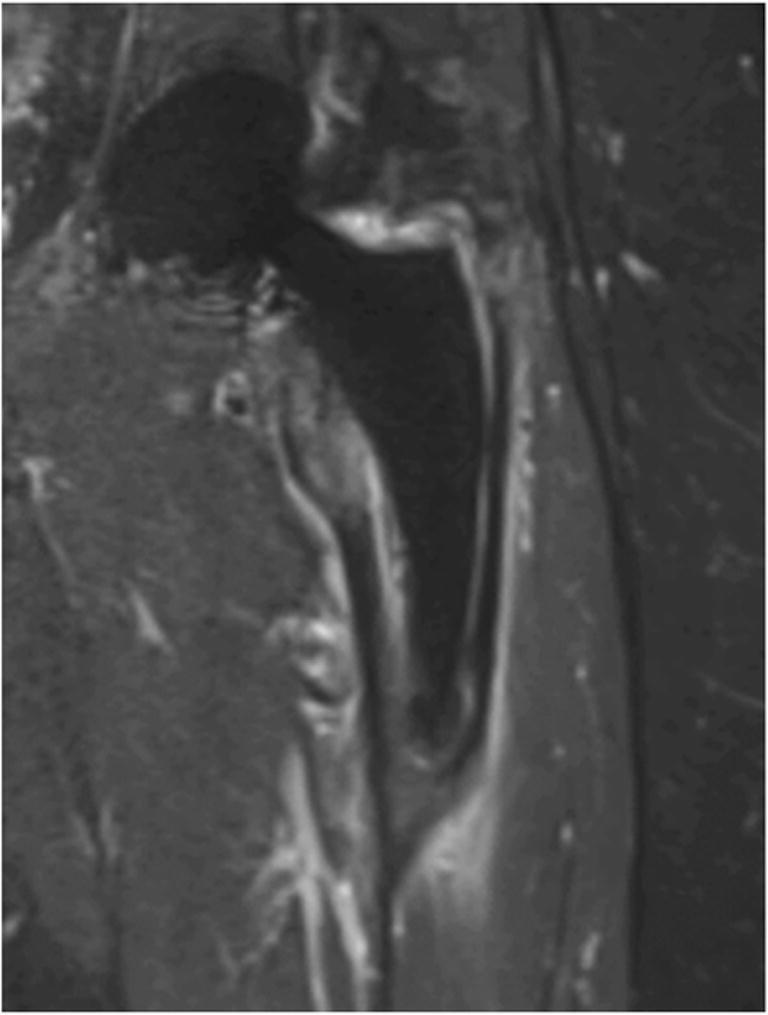
67-year-old female patient with left total hip arthroplasty (THA) on coronal short tau inversion recovery (STIR) image with compressed sensing and slice encoding for metal artifact correction (CS-SEMAC) with a cementless short curved stem (S1). Increased signal between femur and prosthesis stem is seen. This prosthesis was considered loose and this patient was excluded from the study.

This retrospective study included consecutive patients that underwent MRI from February 2015 until October 2019 and was approved by the Zurich cantonal ethics committee. A total of 263 patients with an age above 18 years and with hip prostheses on either one or both sides were included. The following inclusion criteria were applied: availability of plain radiograph for the verification of stem type and hip MRI with an additional axial series over the knee for antetorsion measurements. Patients were operated on and referred to our institution either from the in-house orthopedic department or from an external partner hospital. All imaging was performed at our institution. The following exclusion criteria applied: artifact significantly limiting evaluation for anteversion. Stem loosening was defined as ≥ 2 mm of increased signal between femur and prosthesis stem or between femur and cement in the case of a cemented stem on fluid sensitive sequences. An example of suspected stem loosening is given in Fig. 1. Loosening may lead to secondary rotation of the stem in the femur and falsify the initial postoperative femoral antetorsion.

### Study design

**Fig. 2:**
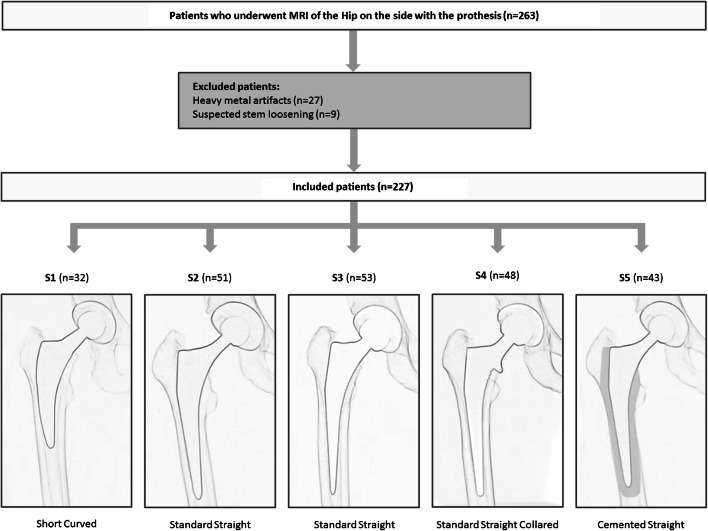
Flow diagram of the inclusion of patients and a schematic drawing of the different prosthesis stems

The study design is presented in Fig. 2. Five different patient groups according to five different stem types from different manufacturers with or without cementation were analyzed; stem 3 and stem 4 were from the same manufacturer but had different designs. The groups were labeled stem 1 to stem 5 (S1–S5). Stem 1 Fitmore, Zimmer Biomet Holdings Inc., Warsaw, Indiana (S1, *n* = 32), was cementless and short curved. Stem 2 Quadra, Medacta Group SA, Castel San Pietro, Switzerland (S2, *n* = 51), and stem 3 Corail, DePuy International Ltd., Leeds, England (S3, *n* = 53), were cementless and standard straight. Stem 4 Corail Collared, DePuy International Ltd., Leeds, England (S4, *n* = 48), was collared cementless standard straight and stem 5 (S5, *n* = 43) was cemented straight from two different manufacturers: Avenir, Zimmer Biomet Holdings Inc., Warsaw, Indiana, and Quadra, Medacta Group SA, Castel San Pietro, Switzerland. For method validation, the accuracy of femoral antetorsion measurements in MRI was tested against CT, which is the current gold standard. In 30 cases, femoral antetorsion was measured in CT and MRI in the same patients. Measurements of the femoral antetorsion in the CT were started 2 months after the MRI measurements were completed to reduce recall bias by the two readers. Femoral antetorsion was measured as described below.

### Imaging

All patients underwent MRI of the hip on a 1.5T Magnetom Avanto-fit system (Siemens Healthcare, Erlangen, Germany). For all hip MRI examinations, patient positioning is standardized. After placement of the patient in a supine position, a tape is carefully put around the feet of the patient in order to fix the position of the lower extremity in a slight internal rotation of the hip during the scan. The MR protocol was specially tailored for THA (Table 1) and included strictly axial T1-weighted imaging with high bandwidth for metal artifact reduction at the level of the joint. To assess femoral antetorsion, a fast T2-weighted, strictly axial haste sequence at the level of the knee was used. No manual tilting of these sequences was performed during imaging acquisition. A body matrix phased-array surface coil and the integrated spine matrix coil were used for imaging of the hip. For the knee, the integrated spine matrix coil was used.

**Table 1:** Scanning parameter for THA protocol

Parameter	Coronal STIR CS-SEMAC	Axial STIR WARP	Coronal T2 high-bandwidth	Axial T1 high-bandwidth	Sagittal T1 high-bandwidth	Axial T2 haste (knee)
TR/TE (ms)	4220/36	4000/31	4000/58	669/8.6	627/7.3	1400/93
ETL	9	11	15	3	3	154
NSA	1	3	2	2	2	1
Number of slices	25	27	20	29	31	10
Section thickness (mm)	4	7	4	6	4	5
Spacing (mm)	4	8.75	6	8.4	4.4	6.5
Matrix	256 × 205	384 × 269	512 × 282	512 × 410	320 × 320	256 × 256
FOV (mm^2)^	280 × 280	189 × 189	220 × 220	210 × 210	200 × 200	240 × 240
Bandwidth (Hz/pixel)	500	450	390	425	435	700
Slice encoding steps	19/13	–	–	–	–	–
TA (min:s)	06:19	03:56	02:28	02:17	01:59	00:14

Detailed 1.5T MRI protocol optimized for metal artifact reduction. *CS* compressed sensing, *ETL* echo train length, *FOV* field of view, *NSA* number of signal averages, *SEMAC* slice encoding for metal artifact correction, *STIR* short τ inversion recovery, *TA* acquisition time, *TE* echo time, *TR repetition time*

Evaluation in respect to potential shaft loosening was done on coronal short tau inversion recovery (STIR) image with compressed sensing and slice encoding for metal artifact correction (CS-SEMAC) [13–15], as well as an axial high-bandwidth STIR sequence with an optimized inversion pulse [16].

CT scans were performed on a Somatom Edge Plus system (Siemens Healthcare, Erlangen, Germany). Patient positioning was similar to positioning in the MRI with lower extremities in fixed, slight internal rotation. The following scanning and reconstruction parameters were used: voltage, 120 kV using 4D care dose; pitch, 0.8; slice thickness, 1.5 mm; increment 1.0 mm; FOV 200 × 200 mm^2^ at the level of the hip and the knee (femoral condyles to joint space). For reconstruction, Kernel Br51 and Strength/Safire 3 were used.

### Image evaluation

Two fellowship-trained musculoskeletal radiologists with 6 and 7 years of experience in musculoskeletal radiology independently evaluated all patients on our institution’s Merlin PACS (Phoenix-PACS GmbH, Freiburg, Germany).

**Fig. 3:**
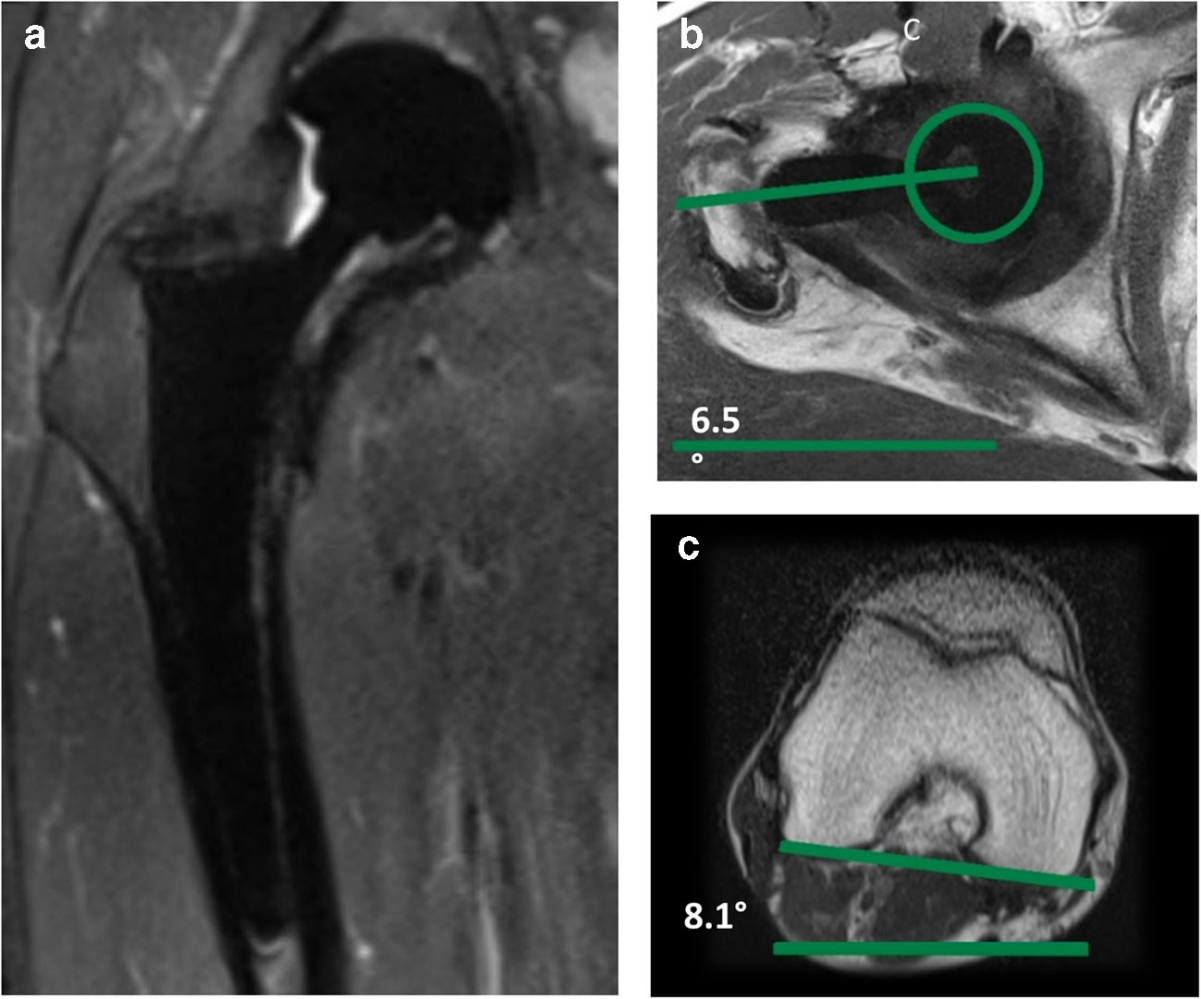
50-year-old male patient with right total hip arthroplasty (THA) on metal artifact suppressed MRI. **a** Coronal short tau inversion recovery (STIR) image with compressed sensing and slice encoding for metal artifact correction (CS-SEMAC) visualizing the THA with a cementless standard straight stem (S2). **b** Axial T1-weighted image with high bandwidth at the level of the hip joint with an angle aligned along the neck of the femoral component (green line). **c** Axial T2-weighted image at the level of the distal femur with a tangent aligned to the posterior femoral condyles (green line). The femoral antetorsion is the combination of the angles in **b** and **c**, which was 14.6° in this patient

For angle measurements, the PACS toolbox was used. First, the head of the prosthesis was delineated with a circle on multiple axial sections simultaneously for defining the center of the head on the whole image stack. At the level of the prosthesis neck, the midpoint between the anterior and posterior contour served as the second reference point. Subsequently, an angle measurement was performed between the proximal and distal femur as follows: (i) proximal line through the prosthesis neck and the center of the prosthesis head and (ii) distal line, which was the posterior condylar tangent of the knee on the second imaging series [17]. An example is given in Fig. 3. CT measurements of femoral antetorsion were done similar to MRI measurements.

### Statistics

Statistical analysis was performed on SPSS version 21.0 (IBM Corp, Armonk, NY) and MedCalc version 17.6 (MedCalc Software bvba, Ostend, Belgium). For continuous data, general descriptive statistics were reported as means and standard deviation (SD). Normal distribution of the measured angles, patient age, and sex was evaluated with the Kolmogorov-Smirnov test; for statistically significant differences in femoral antetorsion, the *t* test for independent samples was applied. The Kruskal-Wallis test checked for differences in age, gender, and occurrence of retrotorsion. Postoperative torsional variability for each group is represented by the SD; we used the *F*-test to check the SD in the cementless and the cemented group for statistically significant differences. In all tests, a *p* value of < 0.05 was considered to represent statistical significance.

The two-way random-effects intraclass correlation coefficient (ICC) was applied for inter-reader agreement, whereas ICC values > 0.75 were considered good agreement, and > 0.9 as very good [18].

## Results

### Included patients

A total of 263 consecutive patients were reviewed for inclusion in the study. In 27 cases, metal artifacts at the level of the head of the prosthesis were very heavy and it was not possible to delineate the femoral head precisely. Inaccurate localization of the center of the femoral head would lead to inaccurate measurement of the femoral antetorsion and therefore led to exclusion. Suspected stem loosening led to the exclusion of another 9 patients, resulting in a final set of 227 cases.

### Demographics

Distribution of age and gender among the different groups is given in Table 2. The median age of the 227 patients was 64.0 years (range 30.0–87.0 years, interquartile range (IQR) 55.0–74.0). The overall age was not normally distributed; this variable was considered non-parametric. Using the Kruskal-Wallis test, age in the cemented group S5 was significantly higher compared with the non-cemented S2 (*p* = 0.001), S3 (*p* = 0.031), and S4 (*p* < 0.001).

**Table 2:** Distribution of age and gender among stem groups S1–S5

Group	Age (median)	Age (IQR)	Gender (m/f)
S1 short curved	66.0 years	54.5–74.0	19 m, 13 f
S2 standard straight	61.0 years	50.3–70.8	30 m, 21 f
S3 standard straight	64.0 years	56.8–73.0	22 m, 31 f
S4 standard straight collared	58.5 years	54.0–67.5	23 m, 25 f
S5 cemented straight	72.0 years	66.0–79.0	9 m, 34 f

Age is not normally distributed and considered non-parametric. *IQR* interquartile range, *m* male, *f* female

Overall, there were 103 males and 124 females included. There were significantly more females in S5 compared with those in S1 (*p* < 0.001), S2 (*p* = 0.001), S3 (*p* < 0.005), and S4 (*p* < 0.005).

### Comparison between CT and MRI femoral antetorsion measurements

Cases from all subgroups were included as follows: stem 1, 7 cases; stem 2, 7 cases; stem 3, 6 cases; stem 4, 6 cases; and stem 5, 4 cases. Mean time between CT and MRI examination was 7.97 months. In 14 cases, CT was prior to the MRI examination; in 15 cases, MRI was prior to the MRI examination; in one case, MRI and CT were performed on the same day.

ICC was calculated between CT and MRI for reader one and reader two. For both, ICC was very good with an ICC of 0.98 (0.96; 0.98) and 0.91 (0.79; 0.96), respectively.

### Femoral antetorsion analysis

The ICC was very good with a value of 0.98 (0.973; 0.984), and the measured femoral antetorsion angles of both readers were averaged. Angles were normally distributed; this variable was considered parametric.

**Fig. 4:**
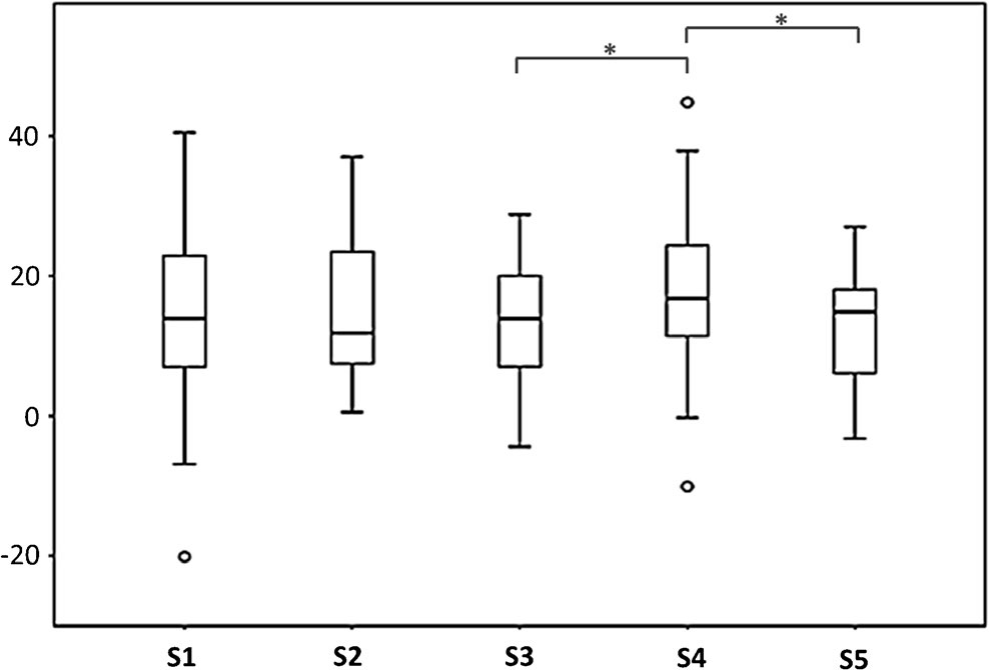
Box plots of femoral antetorsion for stem types S1–S5. Lower and upper edge of box indicates interquartile range, horizontal line inside box indicates median. Whiskers represent lower and upper quartiles, respectively. Outliers are indicated by circles. Asterisks indicate statistically significant differences between groups

Results, standard deviation, and range are shown in Table 3 and Fig. 4. Antetorsion was highest in the collared, cementless standard straight group (S4) with a mean value of 18.1° and lowest in the cemented straight stem group (S5) with a mean value of 12.7°. The measured angles were significantly different between S4 and S5 (*p* = 0.007). The cementless short curved (S1) and both cementless standard straight (S2 and S3) were below S4 and above S5, with mean values of 14.8°, 14.1°, and 13.3° with no statistically significant difference. A significant difference was found between S3 and S4 (*p* = 0.012).

**Table 3:** Femoral antetorsion angles according to stem groups S1–S5

Group	Antetorsion	Standard deviation	Range
S1 short curved	14.8°	± 10.0	0.6°; 37.1°
S2 standard straight	14.1°	± 12.2	− 20.2°; 40.5°
S3 standard straight	13.3°	± 8.4	− 4.4°; 28.8°
S4 standard straight collared	18.1°	± 10.5	− 10.1°; 44.8°
S5 cemented straight	12.7°	± 7.7	− 3.3°; 27.0°

Positive femoral antetorsion angles are given as positive values; femoral retrotorsion angles are given as negative values

**Fig. 5:**
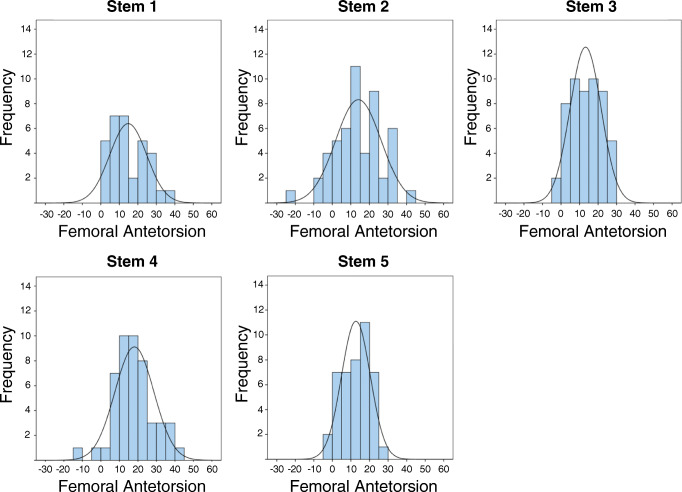
Distribution of femoral antetorsion for each stems S1–S5. Postoperative measured antetorsion angles were grouped in five-degree steps for this figure. A normal distribution curve is overlaid

In all prosthesis types, the range and the SD were high. Figure 5 shows the distribution of the femoral antetorsion for the different stems. For S1, 9 of 32 (28.1%) stems were positioned in the target range of 10° to 20° during surgery; S2, 15 of 51 (29.4%); S3, 19 of 53 (35.8%), S4, 20 of 48 (41.7%); and S5, 19 of 43 (38.8%). Except for S1, positioning in retrotorsion occurred: S2, 6 of 51 (11.8%); S3, 2 of 53 (3.8%); S4, 2 of 48 (4.2%); and S5, 1 of 43 (2.3%). Positioning in retrotorsion was statistically different among the different groups (*p* < 0.001). A comparison of the combined SD between all cementless stems (S1–S4) and the cemented group (S5) was significantly different (*p* = 0.019). SD was highest in one of both cementless standard straight (S2, SD = 12.2) and lowest in the cemented group (S5, SD = 7.7), also with a significant difference (*p* = 0.006). Significant differences in SD were seen for both standard straight stems (S2 and S3) (*p* = 0.018).

## Discussion

This is the first study reporting the postoperative range of femoral antetorsion in patients with THA for different stem designs. Femoral antetorsion was first described as a characteristic of normal anatomy in 1868 by Wolff [19]. There is a natural evolution of the femoral antetorsion during life, with highest antetorsion found after birth (mean, 31.1° ± 8.9) and a gradual decrease towards adolescence (mean 15.4 ± 7.6° at the age of 16) [20]. Normal femoral antetorsion was described with 9.7° to 12.8° [3, 21, 22]. Different methods for the measurement of the femoral antetorsion angle exist at CT and MRI and vary slightly depending on which method is used [23–26]. Measurements of postoperative antetorsion can be done precisely in postoperative MRI with metal artifact suppression which is represented by the very good interobserver agreement between CT and MRI in our study.

In preparation for hip surgery, imaging is required. Usually computer-assisted 2D or 3D planning software based on radiographs is employed [27], and recently CT-based applications are available [28].

For correct postoperative alignment, the combined anteversion approach is an established technique. After the capsulotomy, osteotomy of the neck is performed according to the surgical planning and the femoral head is removed. For acetabular preparation, reamers of increasing size are used until reaching the planned size. Then, a pressfit cup is impacted while paying attention to adequate inclination (40° ± 10°) [7] and anteversion (20°–30°). The femoral canal is then reamed with broaches of different sizes. During broaching, the antetorsion was visually controlled to target 15° [11]. After reaming the definitive size, either a non-cemented, pressfit stem is introduced or a cemented stem using bone cement is implanted.

The combined version of both, the cup and the stem, is important for the stability of the total hip arthroplasty. The position of both the implants should be within the so-called safe zone (25–50°) [11] to minimize the risk for postoperative dislocation. However, non-cemented pressfit stems have to be canal-filling. As a consequence, the individual bony anatomy may impose a suboptimal antetorsion during implant placement. In contrast, cementation of the femoral stem is known to give more freedom to customize antetorsion because the cement—before it hardens—allows better manual control of the stem. Postoperative dislocation is reported between 0.2% and 10% per year, whereas the head-to-neck ratio is of special importance [7, 9]. Postoperative impingement can occur in patients with THA, such as femoroacetabular impingement [5], iliopsoas impingement [29], or ischiofemoral impingement which especially can result in a reduced range of motion [30].

### Stem design as an influencing factor

This study confirmed that there is a wide variety in postoperative femoral torsion in patients with THA.

The measured antetorsion in the short curved (S1, 14.8° ± 10.0°) and both collarless standard straight stems (S2, 14.1° ± 12.2° and S3, 13.3° ± 8.4°) were only slightly elevated compared with the femoral antetorsion in healthy individuals, described by Sutter et al. [3] with similarities in SD (12.7°–13.5° ± 9.8°–10.8°). Antetorsion values in the collared stem group (S4, 18.1° ± 10.5°) were explicitly higher compared with all other groups with statistical significance to the cemented stems (S5) and to one of both cementless collarless standard straight stems (S3). Remarkably, S3 and S4 are of the same manufacturer, only differing in the presence or absence of a collared neck. When introduced into the femur, both stem types (S3 and S4) are likely to have equal physical properties and behave in a similar way, the collar is unlikely to directly influence antetorsion during the introduction process, and placement in elevated antetorsion in S4 cannot be attributed to stem design below the collar. Still, there is a significant difference in femoral antetorsion between both groups. Before the introduction process of the stem into the femur begins, the surgeon visually assesses the antetorsion angle and targets an angle of 15° antetorsion. Intraoperative estimation of stem torsion on a visual basis alone is known to be difficult even for experienced surgeons. In a recent study intraoperative, visual assessment led to the overestimation of femoral antetorsion. Estimated stem torsion was an average of 7.3° higher than CT measurement [31]. Based on our data, we suspect that during surgery the presence of the collar might impair the surgeon’s intraoperative angle estimation and leads to intraoperative visual miscalculation which results in increased postoperative antetorsion.

In S2, there was the highest frequency of postoperative retrotorsion (11.8%) and the highest absolute retrotorsion (− 20.2°), whereas no retrotorsion could be observed for the short curved stem type (S1). During surgery, when the stem is introduced into the femur, torsion towards misalignment starts after the stem has locked and is further introduced. Exceptional malpositioning in retrotorsion might be less likely in a short curved stem because the short stem is put less deep into the femur compared with a long shaft.

For orthopedic surgeons, knowledge of stem specific properties may help to achieve a better surgical outcome. Based on our data, the collared standard straight stem (S4) is more likely to be placed in elevated antetorsion compared with other groups. Future studies may be able to demonstrate whether the insertion procedure for the S4 stem can be modified to achieve a more physiologic femoral antetorsion.

### Cementation as an influencing factor

In the group with the cemented stem (S5), the postoperative femoral antetorsion with a mean of 12.7° is similar to the mean femoral antetorsion in healthy individuals described previously [3]. The standard deviation of femoral antetorsion in cemented stems was lowest and was statistically significantly lower compared with the combined standard deviation of all cementless stems. Positioning in retrotorsion was uncommon (2.3%). A likely explanation is that the cement—while still liquid—allows a certain degree of rotation during and even after complete stem placement which results in a more precise placement. This finding is consistent with the hands-on experience of the surgeons at our institution.

Due to the better control over postoperative femoral antetorsion, we believe that the intraoperatively targeted angle was reached to a higher degree and might be a closer approximation to an ideal postoperative situation compared with the other groups. Statistically significant differences were observed between S4 and S5; we think this is attributed to the rather low standard deviation in the cemented group, which made this difference statistically significant.

In our study, patients with a cemented prosthesis were older compared with the cementless prosthesis: This is a finding in accordance with the clinical indications of hip replacement [32]. In this study, design patient age is most likely not a confounding factor.

### Limitations

This study has the following limitations: Preoperative femoral antetorsion was not known in the included patients, due to the retrospective study design. Although the included patients were all operated by specialized hip surgeons, there was no subgroup analysis for individual surgeons. In group 5, the cemented standard straight design included stems from different manufacturers due to the limited availability of cases from one single manufacturer. There were not an equal number of patients in each group; especially in group 1, there were fewer patients compared with groups 2–5. Still, in this group, the SD lies within the range of the other groups and a statistical effect might be negligible. Method validation through antetorsion measurement in MRI and CT was done in a subset of patients.

## Conclusions

This is the first study reporting the postoperative range of femoral antetorsion in patients with THA for different stem designs. In short, our study showed that prosthesis design seems to impact the postoperative femoral antetorsion. Antetorsion was highest for cementless collared stems and lowest for cemented stems. The cemented stems demonstrated the lowest variability and a low rate of retrotorsion, suggesting the lowest rate of inadvertent malrotation during implant placement.
